# Eucaloric Balanced Diet Improved Objective Sleep in Adolescents with Obesity

**DOI:** 10.3390/nu13103550

**Published:** 2021-10-10

**Authors:** Oussama Saidi, Emmanuelle Rochette, Giovanna Del Sordo, Éric Doré, Étienne Merlin, Stéphane Walrand, Pascale Duché

**Affiliations:** 1 Laboratory Impact of Physical Activity on Health (IAPS), Toulon University, F-83000 Toulon, France; oussama.saidi@univ-tln.fr (O.S.); e_rochette@chu-clermontferrand.fr (E.R.); giovanna.del@hotmail.fr (G.D.S.); 2 Laboratory of Adaptations to Exercise under Physiological and Pathological Conditions (AME2P), Clermont Auvergne University, F-63170 Clermont-Ferrand, France; eric.dore@uca.fr; 3 Regional Center for Human Nutrition (CRNH Auvergne), F-63000 Clermont-Ferrand, France; 4 Department of Pediatrics, Clermont-Ferrand University Hospital, F-63000 Clermont-Ferrand, France; e_merlin@chu-clermontferrand.fr; 5 INSERM, CIC 1405, CRECHE Unit, CHU Clermont-Ferrand, Clermont Auvergne University, F-63000 Clermont-Ferrand, France; 6 INRAE, UNH, CHU Clermont-Ferrand, Clermont Auvergne University, F-63000 Clermont-Ferrand, France; stephane.walrand@inra.fr

**Keywords:** obesity, polysomnography, energy balance, energy intake, evening meal, youth

## Abstract

Background: A better understanding of the influence of energy balance on sleep in adolescents, particularly those with obesity, could help develop strategies to optimize sleep in these populations. The purpose of this study was to investigate sleep under ad libitum-vs-controlled diets adjusted to energy requirement (eucaloric) among adolescents with obesity and their normal weight controls. Methods: Twenty-eight male adolescents aged between 12 and 15 years, *n* = 14 adolescents with obesity (OB: BMI ≥ 90th centile) and *n* = 14 normal weight age matched controls (NW), completed an experimental protocol comprising ad libitum or eucaloric meals for three days, in random order. During the third night of each condition, they underwent in home polysomnography (PSG). Results: An interaction effect of energy intake (EI) was detected (*p* < 0.001). EI was higher during ad libitum compared to the eucaloric condition (*p* < 0.001) and in OB compared to NW (*p* < 0.001) in the absence of any substantial modification to macronutrient proportions. Analyses of energy intake distribution throughout the day showed a significant interaction with both a condition and group effect during lunch and dinner. Sleep improvements were noted in OB group during the eucaloric condition compared to ad libitum with reduced sleep onset latency and N1 stage. Sleep improvements were correlated to reduced EI, especially during the evening meal. Conclusion: Simply adjusting dietary intake to energy requirement and reducing the energy proportion of the evening meal could have therapeutic effects on sleep in adolescents with obesity. However, positive energy balance alone cannot justify worsened sleep among adolescents with obesity compared to normal weight counterparts.

## 1. Introduction

Striking data from all over the world has reported commonly poor sleep patterns among adolescents [1,2]. A problem compounded by an array of endogenous and exogenous factors forming the so-called “Perfect Storm” of both altered sleep duration and quality [3]. Furthermore, obesity has been associated with sleep disturbances through several pathogenetic pathways [4]. Several clinical studies underlined how sleep affects both components of energy balance [5,6]. Although the effect on energy expenditure remains uncertain, a growing body of evidence demonstrates that sleep alteration increases energy intake with a more pronounced craving for energy-dense foods through numerous hormonal, neuroendocrine, cognitive, metabolic, and behavioral pathways [7,8,9] and these findings have also been noted in youth [10,11]. However, it appears that a potential bidirectional effect links sleep to energy balance and that in turn both nutrition and physical activity could exert ongoing feedback on biological clock and sleep physiology.

Although the effect of physical activity on sleep is well documented in both adult and young subjects [12,13], the effect of nutrition has been disregarded for a long time. However, a growing interest in this topic has emerged over the last decade and several reviews have been published [14,15,16,17]. Cross-sectional studies found numerous associations between dietary patterns and sleep [18,19,20]. However, fewer studies included objective sleep measurement. Spaeth et al., (2017) reported that in healthy adults, dietary intake was associated with sleep measured by polysomnography (PSG). Greater protein intake and lower carbohydrate intake were associated with more time spent in rapid eye movement (REM) sleep, while higher fiber was associated with increased slow wave sleep (SWS) [21]. In another study, higher nocturnal fat intake was associated with increased wake after sleep onset (WASO) and reduced sleep efficiency (SE) [22]. Besides these descriptive observational studies, some clinical trials attempt to report how dietary manipulation may affect sleep staging and quality [23,24,25]. However, the overwhelming majority of these studies were conducted in laboratory settings among healthy adults and potentially good sleepers who may be resistant to the effects of nutrition on sleep. Moreover, physical activity was not assessed during these interventions and may represent a potential confounder.

Adolescents, especially those with obesity, seem to be vulnerable to sleep disturbances [26]. Unfortunately, few interventional lifestyle studies, intended to improve sleep, were conducted among these populations. Yet, both nutrition and physical activity could offer basic options to improve sleep during adolescence. This is of significant importance especially for those with obesity since the latest recommendations have encouraged the consideration of sleep in the management of pediatric obesity [27]. A more comprehensive understanding of how energy balance affects sleep in these populations would be of considerable interest in the development of future dietary strategies promoting better sleep and effective weight loss intervention. Therefore, in this study we sought to examine objectively measured sleep under two different conditions (1) controlled balanced diet adjusted to energy requirement (eucaloric), and (2) diet offered ad libitum among adolescents with obesity and their age-matched normal weight peers. In other words, we intended to examine if an adolescent with obesity in neutral energy balance and balanced macronutrient proportions would have better or worse sleep quality than a normal weight adolescent in positive energy balance and vice versa. We hypothesize that a eucaloric balanced diet would improve sleep quality in both groups. However, given that adolescents with obesity may experience more sleep disturbances, a greater improvement was expected in this group according to the ceiling and floor effect.

## 2. Materials and Methods

### 2.1. Subjects

A total of 28 male adolescents aged between 12–15 years, and over Tanner stage 3, either with obesity (*n* = 14) (BMI ≥ 90th centile) recruited through the Pediatric Obesity Centre (Tza Nou, La Bourboule, France) or normal weight age matched controls (*n* = 14) recruited in collaboration with the Pediatric Department, CHU (Clermont-Ferrand, France) participated in this study. No participants suffered from any diagnosed major sleep disorders, e.g., narcolepsy, obstructive sleep apnea (OSA), or obesity comorbidities, e.g., diabetes, or used any medication or therapy that may interfere with sleep outcomes, e.g., antidepressants or benzodiazepines. Moreover, they were screened for depression using the Kutcher Adolescent Depression Scale (KADS) (LeBlanc et al., 2002). All included participants presented a score below 6 in this questionnaire indicating a low risk of depression. Adolescents with obesity were living in “Tza Nou” center (Obesity Treatment Center for Children and Adolescents). Normal weight peers were recruited from a boarding school to ensure comparable living conditions between groups. The investigators traveled to both sites with the laboratory equipment to ensure an ambulatory running of the study. The present study received approval from the relevant Institutional Ethics Review Board (Clinical trial No. NCT04041934). The research protocol was in line with the principles of the Helsinki Declaration. Information and consent forms were distributed to parents and adolescents prior to the study launch.

We underline that although we carefully included adolescents without diagnosed OSA, it turned out that some participants from the OB group (*n* = 4) presented undiagnosed OSA. During the habituation night, they presented an Apnea Hypopnea Index (AHI) of (4–5) episodes·h^−1^. Yet, during the experimental protocol, the value for AHI was mildly >5 episodes·h^−1^ potentially indicating the presence of OSA.

### 2.2. Study Design and Procedure

Participants were assessed for anthropometric and body composition. They filled in questionnaires in paper format regarding their circadian typology: the Horne–Östberg Morningness–Eveningness Questionnaire (MEQ), sleep quality: Pittsburgh Sleep Quality Index (PSQI), and sleepiness: Epworth Sleepiness Scale (ESS). One week prior to the launch of experimental sessions, time in bed (TIB) was fixed (from 22:00 to 7:00) in order to avoid the potential effect of an irregular bedtime schedule on circadian rhythm and sleep quality. During this week, participants resting metabolic rate (RMR) was assessed by indirect calorimetry. They wore accelerometers to assess physical activity and energy expenditure. The investigators also equipped participants with portable PSG in order to familiarize them sleeping with the device.

**Figure 1:**
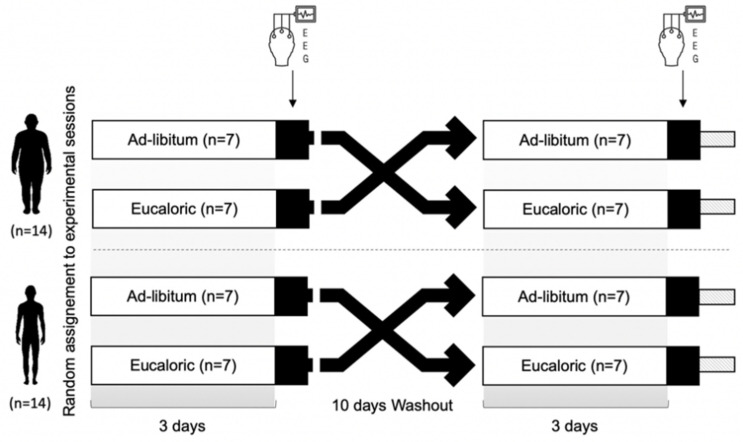
Study design.

Subsequently, adolescents participated in the experimental protocol comprising two three-day sessions in random order, separated by a 10-day washout: one session was eucaloric, and the other ad libitum (Figure 1). A manipulation check was performed to ensure the washout period was effective at taking all participants back to baseline sleep and dietary intake between the two sets of conditions (eucaloric vs. ad libitum). No violations occurred. Physical activity was continuously monitored by accelerometry. In order to reduce biases that might overwhelm the relationship between sleep and dietary intake, participants were asked to maintain the same habitual daily activities as in the previous week and to refrain from any structured vigorous physical activities at least 48 h before the experimental protocol. Moreover, all sessions took part on the same days of the week and no scholastic commitments were held during the sleep assessment nights. The use of electronic media (smartphone, laptop...) in the evening was prohibited and evening bedtime was fixed. During night 3, participants were instructed to go to their room at 21:00. The investigators equipped them with PSG devices and the light was switched off at 22:00. In this manner they were given an ideal time of 9 h for sleep (from 22:00 to 07:00).

### 2.3. Baseline Evaluations

#### 2.3.1. Anthropometry and Body Composition

Barefoot height was measured using a portable stadiometer (TANITA, HR001, Ja-pan). Body mass (BM) was measured using a digital scale (TANITA, BC-545N, Japan). Subsequently, Body Mass Index (BMI) was calculated as follows; BMI = body mass divided by height squared expressed in kg/m^2^. Fat mass, and fat free mass were determined using dual-energy X-ray absorptiometry (QDR4500A scanner, Hologic, Waltham, MA, USA).

#### 2.3.2. Circadian Typology and Subjective Sleep

The Horne–Östberg Morningness–Eveningness Questionnaire (MEQ) was used to assess circadian typology based on habitual waking and bedtimes as well as the times of day at which an individual prefers to ‘perform’ [28]. The PSQI was used to evaluate the quality of sleep during the last month based on 19 items covering seven clinically-relevant components of sleep difficulties. Each item is weighted on a scale, and a global score of sleep quality is calculated by adding up the seven component scores, producing an overall PSQI score ranging from 0–21, where a score > 5 indicates poor sleep quality [29]. The Epworth Sleepiness Scale (ESS) was used to measure daytime sleepiness [30]. The ESS consists of a simple self-report questionnaire with eight questions and responses on a 4-point Likert scale (0–3). An ESS score > 10 indicates excessive daytime sleepiness and the possibility of a high risk of underlying sleep disorders.

#### 2.3.3. Resting Metabolic Rate

Resting metabolic rate (RMR) was measured in the morning, in a fasted state, using indirect calorimetry (Metamax 3B portable gas analyzer, Cortex, Leipzig, Germany). Before each test, the equipment was calibrated in accordance with the manufacturer’s recommendations. Participants were placed in a supine position in a thermoneutral environment (22–25 °C room temperature) for 45 min before starting the measurements. After achieving a steady state, O_2_ consumption and CO_2_ production, standardized for temperature, barometric pressure, and humidity, were recorded at 1 min intervals for 20–45 min and averaged over the whole measurement period. Resting metabolic rate (in kcal/day) and respiratory quotient (ratio of CO2/O_2_) were calculated thereafter.

#### 2.3.4. Habitual Physical Activity and Energy Expenditure

Accelerometers are a non-intrusive and cost-effective tool that provide accurate and reliable measurement of physical activity and energy expenditure in youth under free living conditions. ActiGraph accelerometers (GT3X+, Actigraph LLC, Pensacola, FL, USA) were initialized to record data in 60-s epochs and worn for 3-consecutive days (one week prior to experimental sessions) on the dominant hip as recommended by the manufacturer. The device was only removed when water contact was possible (bathing, swimming). Data were analyzed using the manufacturer’s software (Actilife 6.0, Pensacola, FL, USA). Physical activity was assessed based on the translation of count per min (CPM) into time (min/day) spent at various levels of movement intensity. Thresholds of CPM for sedentary, light, moderate, and vigorous levels of physical activities were defined according to Trost et al., (2011) [31]. Adolescents’ energy expenditure was individually calculated based on RMR and daily metabolic equivalent of task values derived from the accelerometer’s raw data.

### 2.4. Experimental Sessions

#### 2.4.1. Dietary Intervention

All meals conformed to the adolescents’ tastes as determined by a food questionnaire completed at baseline (Table S1). Disliked items were excluded in order to limit leftovers and prevent uncontrolled snacking outside planned meals during the eucaloric session. Top rated items were avoided to limit excessive intake and reflect habitual dietary patterns during the ad libitum session. Dietary intake during eucaloric sessions was planned by a registered dietician and was not designed to produce weight loss. The diet met the mean estimated energy requirement of adolescent males. Energy requirement was established individually based on subject’s characteristics as well as physical activity and energy expenditure tracked on the previous week with the following macronutrient distribution: 18:52:30 (Protein:Carbohydrates:Fat). Individual dietary intake was calculated in advance and meal trays were prepared for each subject. Energy intake (EI) was distributed as follows: (25% from breakfast, 30% from lunch, 30% from dinner, and 15% from snacks). Participants were encouraged to finish their entire meals. If not, food leftovers were weighed and recorded, then they were subtracted from the initially calculated food intake. During the ad libitum session food was offered as buffet meals and the participants were asked to eat until they were satisfied. Food consumption was weighed and recorded by investigators. Energy intake and macronutrient distribution were calculated using a professional computerized nutrient analysis program (Bilnut 4.0 SCDA Nutrisoft software, France) and Ciqual tables (year-2018 version). Three meals and a snack were offered daily for both sessions at regular time intervals: 7:30 (breakfast), 12:00 (lunch), 17:30 (snack), and 19:00 (dinner). Caffeinated beverages such as colas, coffee, or tea were not allowed during the experimental sessions.

#### 2.4.2. Polysomnography

The Sleep Profiler PSG2 (Advanced Brain Monitoring, Carlsbad, CA, USA) is an ambulatory alternative to laboratory polysomnography approved by the Food and Drugs Administration. The system provides access to 13-channels: electro-encephalography (EEG), electro-oculography (EOG), and electro-myography (EMG) of front-polar sites (AF7-AF8, AF7-Fpz, and AF8-Fpz) allowing the characterization of sleep staging and quality as well as wireless oximetry, nasal pressure/airflow, chest and abdomen respiratory effort, forehead and finger pulse rate, head movement and position, and quantitative snoring. The obtained records were uploaded to the Sleep Profiler portal where automated algorithms were applied to the signals. Auto-staging was performed based on the ratios of the power spectral densities and auto-detection of cortical and microarousals, sleep spindles, and ocular activity [32,33]. After the processing of sleep recordings, an experienced sleep expert reviewed each recording in order to confirm the accuracy of the auto-sleep staging and customize editing if needed. Sleep study featured the following outcomes: total sleep time (TST), sleep latency (SL), wake up after sleep (WASO), sleep efficiency (SE), arousal index, as well as sleep architecture (Wake, N1, N2, N3, REM sleep) according to recommendations of the American Academy of Sleep Medicine (AASM).

### 2.5. Statistical Analysis

Data analyses were performed using SPSS, (Version 26, SPSS, Inc, Chicago, IL, USA) Pre-experimental differences in characteristics between adolescents with obesity and their matched controls with regard to anthropometry, body composition, circadian typology and subjective sleep, as well as resting metabolic rate, physical activity, and energy expenditure were assessed using t-tests. Repeated measures analysis of variance (ANOVA) were performed with a 2 (weight status as between-subject factor) × 2 (conditions as within-subject factor) design, in order to assess differences between groups (obese vs. normal weight) and conditions (ad libitum vs. eucaloric) on each dietary intake variable (total energy intake, %EI from proteins, %EI from lipids and %EI from CHO), energy intake distribution throughout the day (breakfast, lunch, snack and dinner) as well as sleep outcomes (TST, SE, SL, Wake, WASO, REM, N1, N2, N3, and arousals). When significance was obtained, post hoc pairwise comparisons were computed using the Bonferroni method to examine differences between the two conditions (ad libitum vs. eucaloric) for the obese and normal weight groups separately. To test whether the presence of OSA influences the results obtained, additional analyses were conducted while excluding participants with potential OSA. All mentioned effect sizes were obtained using Cohen’s d. Pearson’s correlations were performed to examine the relationships between Δ energy intake and Δ sleep outcomes (ad libitum—eucaloric).

## 3. Results

### 3.1. Subject Characteristics

Twenty-eight adolescents participated in this study, OB (*n* = 14) were adolescents with obesity (BMI ≥ 90th centile) and NW (*n* = 14) were their normal weight age matched controls. Their mean age was 14.0 ± 0.9 years. Detailed characteristics of the two groups are presented in Table 1. The two groups differed in body weight, BMI, and percentage of body fat (all *p* < 0.001). OB presented poorer sleep quality as indicated by PSQI score (*p* < 0.001), as well as higher sleepiness estimated by ESS score (*p* < 0.001). No differences were found in circadian typology (*p* = 0.210). No differences were found in time spent on sedentary, light, and moderate to vigorous physical activity (all *p* > 0.05). However, RMR measured by indirect calorimetry was higher in OB (OB = 2035 ± 364 vs. NW = 1631 ± 106 kcal·day^−1^, *p* < 0.001), which resulted in a higher total energy expenditure (TEE) in OB compared to NW (OB = 2864 ± 369 vs. NW = 2460 ± 148 kcal·day^−1^, *p* < 0.001).

### 3.2. Energy Balance and Macronutrient Intake Outcomes

**Figure 2:**
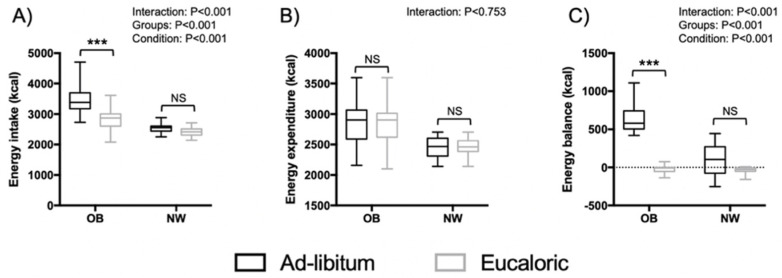
Energy intake (**A**), energy expenditure (**B**) and energy balance (**C**) among OB and NW during ad libitum and eucaloric sessions. OB: adolescents with obesity, NW: normal weight age matched controls, NS: non-significant, ***: significant difference in Post hoc pairwise comparisons (ad libitum vs. eucaloric) with *p* < 0.001.

Figure 2 shows energy intake (EI), energy expenditure (EE), and energy balance (EB) among OB and NW during ad libitum and eucaloric sessions. An interaction effect of EI was detected (*p* < 0.001). Overall, EI was higher during ad libitum compared to eucaloric (*p* < 0.001) and in OB compared to NW (*p* < 0.001). Post-hoc analysis showed a significant increase of EI among OB during ad libitum compared to eucaloric (+653 kcal, *p* < 0.001). However, the increase of EI in NW did not reach statistical significance (+125.8 kcal, *p* = 0.24). Mean values of EE did not differ from pre-experimental measurement. No differences were detected between group nor condition. Thereby, a significant interaction effect of EB was obtained (*p* < 0.001). As for EI, post-hoc analysis showed a significant increase in EB only in OB but not NW group.

Energy derived from each macronutrient is included in Table S2, interaction effects were noted for protein (*p* < 0.001) and CHO (*p* = 0.007). However, macronutrient distribution did not differ between conditions (all *p* > 0.05). The results were similar after excluding subject with OSA from the analyses (Table S3).

### 3.3. Distribution of Energy Intake on the Four Meals

**Figure 3:**
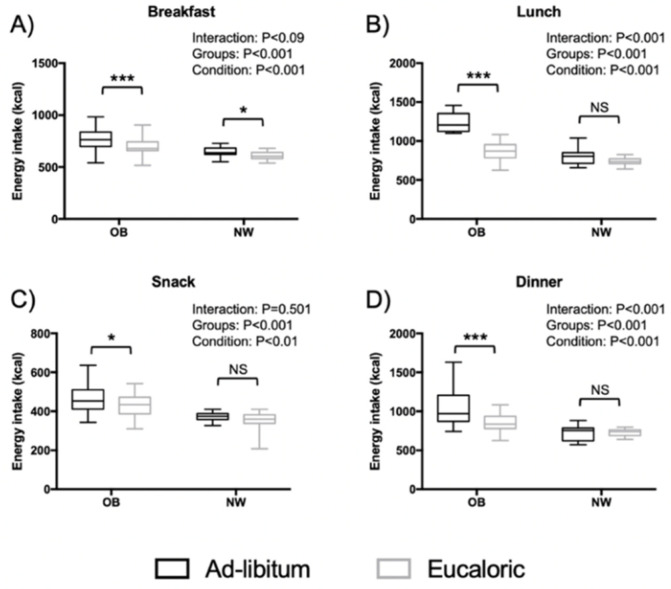
Distribution of energy intake throughout the four meals among adolescents with obesity and their matched normal weight controls during ad libitum and eucaloric sessions. *: significant difference in Post hoc pairwise comparisons (ad libitum vs. eucaloric) with *p* < 0.05. ***: significant difference in Post hoc pairwise comparisons (ad libitum vs. eucaloric) with *p* < 0.001.

Giving that the proportions of all macronutrients were not different between conditions, in Figure 3 we focused on EI distribution throughout the offered four meals among OB and NW between the two sessions. Significant interaction effects were found for EI during lunch and dinner. Consumption of energy intake was higher in OB compared to NW and in ad libitum compared to the eucaloric condition.

### 3.4. Sleep Outcomes

Polysomnography outcomes are shown in Table 2. A significant interaction effect was only detected in N1 sleep (*p* < 0.03). However, trends toward significance were also obtained for SL (*p* < 0.065) with a moderate effect size. There were significant differences between conditions in SL (*p* < 0.001) and N1 (*p* < 0.003). However, no differences in SL and N1 were found between groups. Post hoc tests comparing these outcomes between sessions for each group revealed no differences among NW. However, SL and N1 sleep were significantly reduced in OB adolescents during eucaloric compared to ad libitum sessions. Moreover, in accordance with the subjective sleep evaluation at baseline, poorer sleep quality was obtained in OB adolescents compared to NW during both sessions. This was marked by higher WASO, and Arousals (all *p* < 0.05). All these results are still valid without taking into account subjects with OSA as shown in Table S5.

### 3.5. Correlation of Δ Energy Intake with Δ Sleep Outcomes between Sessions in OB Group

**Figure 4:**
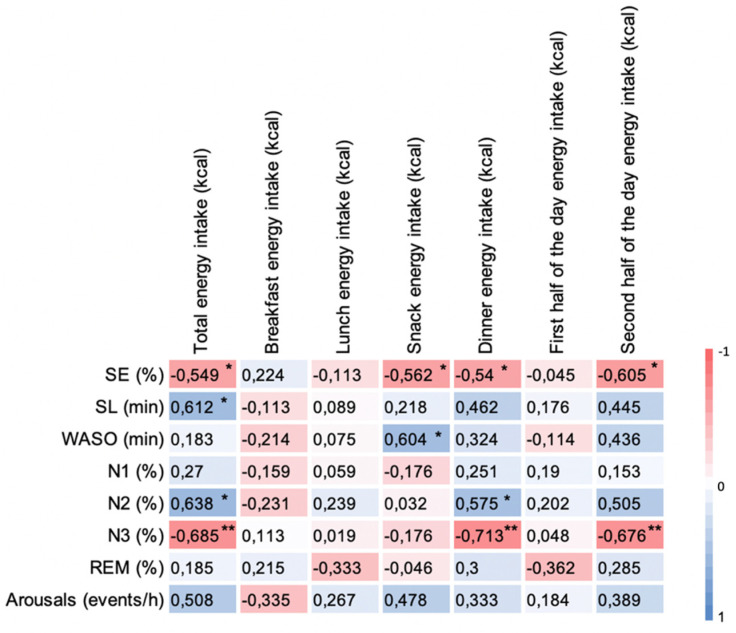
Heatmap representation of the correlations between Δ energy intake and Δ sleep outcomes between sessions (ad libitum—eucaloric) in OB group. Red indicates a negative relationship whereas blue indicates a positive relationship; the darker the color, the higher the Pearson coefficient; *: significant correlation with *p* < 0.05; **: significant correlation with *p* < 0.01.

Figure 4 shows correlations of Δ energy intake with Δ sleep outcomes between sessions in OB group. Increased total energy intake was associated with reduced SE (*p* < 0.05), increased SL (*p* < 0.01), N2 stage (*p* < 0.01), and reduced N3 stage (*p* < 0.01). Increased energy intake during the second half of the day especially during the dinner might be responsible of this effect. Increased energy intake during dinner was associated with decreased SE (*p* < 0.01), N3 stage (*p* < 0.01), and increased N2 stage (*p* < 0.05).

## 4. Discussion

In view of reported poor sleep outcomes in adolescents and based on growing literature connecting sleep to obesity, there is an urgent need for effective non-pharmacological alternatives to improve sleep in this population. Several studies highlighted the adverse effect of poor sleep (duration/quality) on energy balance regulation resulting in increased dietary intakes [6,9]. However, a clearer understanding of the complex interrelationship between sleep and energy balance in the opposite direction is of significant importance particularly in adolescents with obesity since the latest recommendations strongly endorsed the consideration of sleep disturbances in the management of pediatric obesity [27]. In this context, the present study questioned the effect on sleep of adjusting dietary intake according to energy requirements (eucaloric) with a balanced macronutrient distribution compared to ad libitum intake condition designed to reflect habitual dietary patterns. Poorer sleep quality was observed in adolescents with obesity compared to their normal weight peers during both conditions (eucaloric and ad libitum). Only three days of a eucaloric diet were sufficient to allow a reduction of SL and N1 in adolescents with obesity. This result suggests that positive energy balance may exacerbate the effect of obesity on sleep. However, the results also suggest a strong weight status effect on sleep despite feeding condition which means that positive energy balance alone cannot justify worsened sleep among adolescents with obesity compared to normal weight counterparts. No effect was obtained on sleep in normal weight adolescents. However, this might be related to the smaller variation in dietary intake between eucaloric and ad libitum conditions in this group.

The effect of the present dietary interventions on sleep were not surprising. Despite the limited number of clinical trials, an early study by Phillips et al., (1975) reported acute sleep changes in response to dietary manipulation. In fact, lower time spent in slow wave sleep (SWS) was observed following a high-carbohydrate/low-fat diet and higher time spent in SWS upon a low-carbohydrate/high-fat diet compared to the control diet. Moreover, rapid eye movement sleep (REM) was longer with the high-carbohydrate/low-fat diet compared to the low-carbohydrate/high-fat and control diets; while non rapid eye movement sleep (NREM) was shorter with both high-carbohydrate/low-fat and low-carbohydrate/high-fat diets compared to the control diet [24]. Sleep quality changes measured by accelerometry were also notable after 4 days of intervention including isocaloric high-protein or high-fat or high-carbohydrate diets [34]. Others show that carbohydrate intake with high glycemic index was associated with reduced SL [35]. In another study, SWS decreased immediately during the first sleep cycle with a high carbohydrate diet compared to a high fat diet when macronutrient distribution was only manipulated at dinner [25]. However, we highlight that our results documented sleep changes in the absence of any substantial modification in macronutrient proportions. Therefore, the most tangible explanation here could be overall energy intake, or variation of energy consumption distribution on the four meals offered during the day. Correlations between changes in energy intake and changes in SL and N3 suggest that excess energy during the latter meals may be more related to difficulties initiating sleep as well as lighter sleep among adolescents with obesity, but it remains to be demonstrated in normal weight adolescents as the ad libitum condition resulted in a smaller non-significant increase in EI.

Despite the fact that the obtained effect was very modest, our results confirm that excess energy intake, mainly in the latter meals, could alter sleep quality. This is in line with previously published studies. In fact, St-Onge et al., (2016) showed an increase in SL and a decrease in slow wave sleep (Stage N3) following one day of ad libitum feeding compared to those obtained upon 3 days of adapted and balanced diet in 26 normal-weight adults [36]. In another study carried out among 45 adults with obesity and obstructive sleep apnea, a higher caloric intake at night was also associated with higher sleep latency [37]. As detailed above, most studies dealing with the effect of nutrition on sleep focused on macronutrient proportions and their “quality effect”, on sleep. However, the current study highlights that sleep is also sensitive to energy intake variation underlining a “quantity effect”. Excess energy especially in the latter meals appears to exert a negative effect on sleep initiation and structure with more light sleep and less deep sleep. Some clinical evidence suggests that consuming a large morning meal was not associated with obesity while a large evening meal was found to substantially increase the risk of obesity [38]. Moreover, following a 12-week weight loss intervention (isocaloric diet: 1400 kcal per day) among women with obesity, the group consuming a larger breakfast meal lost much more weight and showed better improvement in fasting glucose, insulin, and Homeostatic Model Assessment of Insulin Resistance (HOMA-IR) [39]. That is to say that in spite of the number of calories, early eaters lose more weight and enhance their metabolic profile in comparison with late eaters. The mechanisms behind this temporal effect on weight regulation remain to be further explored. However, it appears that excess energy intake during the latter meals could disrupt the circadian system by inducing a phase shift in the peripheral clock and not the master clock. This circadian misalignment appears to induce adverse effect on metabolism. Fewer studies focused on the effect of evening energy intake on sleep. Driver et al. (1999) reported increased body temperature and suggested that a high energy meal resulted in a long-lasting thermic effect of food that may interfere with sleep episodes. However, no effect of a high energy dinner on sleep compared to a control dinner containing half the amount of calories or even to a fasted condition were obtained. Elevated core body temperature is known to affect sleep onset latency and to decrease slow wave sleep (N3 stage) [40]. However, the aforementioned study was based on the acute effect of one meal and may have been underpowered to detect differences in sleep (*n* = 7). The effect of the digestive system on sleep remains poorly understood and should be further investigated. However, a decrease in the gastrointestinal tract activity from sleep stage N1 to N3 has been already reported [41]. Furthermore, previous studies showed that large meals expand the stomach and increase upward pressure against the lower esophageal sphincter [42]. Therefore, along with the thermic effect of food, mechanical stimulation of the gastrointestinal tract as well as heartburn symptoms near to bedtime could also be responsible for sleep alteration in response to larger energy intake during the latter meals. Finally, we emphasize that the timing of energy intake was restricted in this study. Thus, the effect of delayed meal timing on sleep in patients with obesity needs to be addressed in future studies.

### Limitations, Strengths, and Future Research Directions

This study was strengthened by combining objective assessments of energy balance and sleep in youth under naturalistic free-living settings. However, as reported EI variations between ad libitum and eucaloric sessions were only significant in adolescents with obesity. Our initial hypothesis of better improvement of sleep quality in adolescents with obesity during eucaloric session was only based on celling effect of nutritional intervention on sleep. We actually expected a comparable increase (but lower than that observed in OB group) in EI in the two groups as previously reported in adults [43]. No study to our knowledge has compared the nutritional response between normal weight adolescents and those with obesity over 24 h. However, previous studies have reported similar nutritional response in both groups during ad libitum lunch and dinner [44]. Even if we failed to obtain a significant increase in EI in normal weight adolescents, this result may underline lower loss of control overeating in NW group compared to OB. The choice of this protocol was initially intended to reflect habitual dietary intake in comparison with eucaloric condition. However, eating behavior traits of participants, as well as the inclusion of subjects with obesity taking part in a multidisciplinary weight loss program may also have exaggerated the increase of EI in OB group. We underline though that the ad libitum sessions were organized identically with the same food items in both groups according to Thivel et al. (2016) [45]. On the other hand, although we carefully included adolescents without diagnosed OSA, it turned out that some participants from the OB group (*n* = 4) presented undiagnosed OSA. However, the study results were checked, and no major statistical differences resulted from the inclusion of these participants (Table S3–S5). This could be explained by the mildness of the disease (AHI < 7 episodes·h^–1^).

The majority of previous studies exploring the effect of nutrition on sleep did not consider physical activity. We emphasize that future research should systematically assess physical activity during the intervention, giving the major impact that this behavior plays on metabolism and sleep regulation, even in the short term [46]. The current study opens the horizons for future research investigating the effect of energy balance (both EI and EE) on sleep. It would be interesting to examine the effect of positive, neutral, or negative energy balance on sleep and determine the effect of weight status per se on this relationship. Furthermore, determining if a differential effect on sleep would result from an energy deficit obtained by decreasing energy intake compared to that of increasing energy expenditure would also be interesting. Future studies should also examine if an interaction effect of nutrition and exercise on sleep exist. Finally, the effect of temporal variation in excess energy intake on sleep found in this study brings a new insight to the potential therapeutic effect of chrono-nutrition on sleep in adolescents.

## 5. Conclusions

Reduced energy intake during the eucaloric condition compared to ad libitum feeding for only three days improved sleep quality by decreasing SL and N1 stage. This effect was only obtained in adolescents with obesity given that normal weight adolescents didn’t increase their energy intake between the two conditions.

## Figures and Tables

**Table 1:** Descriptive characteristics, subjective sleep quality, daytime sleepiness, and perceived stress of the sample population.

	OB Mean (SD)	NW Mean (SD)	*p* Value
Anthropometry & body composition			
Age (year)	14.0 (0.9)	14.0 (0.9)	-
Height (cm)	163.0 (11.7)	164.7 (7.8)	0.635
Weight (kg)	88.7 (23.8)	56.27 (6.14)	<0.001
BMI (kg·m^−2^)	33.1 (7.1)	20.71 (1.58)	<0.001
FM (%)	36.9 (5.7)	14.0 (3.2)	<0.001
FFM (kg)	53.4 (12.3)	48.2 (4.7)	0.164
Circadian typology and subjective sleep			
Circadian typology (MEQ_score_)	29.7 (3.4)	32.9 (5.9)	0.210
Sleep quality (PSQI_score_)	8.4 (3.0)	4.2 (1.9)	<0.001
Sleepiness (ESS_score_)	9.3 (3.8)	4.3 (2.0)	<0.001
Physical activity, RMR and TEE			
Sedentary (min·day^−1^)	441 (46)	436 (62)	0.667
Light (min·day^−1^)	240 (36)	235 (43)	0.603
MVPA (min·day^−1^)	50 (27)	63 (39)	0.454
RMR (kcal·day^−1^)	2035 (364)	1631 (106)	<0.001
TEE (kcal·day^−1^)	2864 (369)	2460 (148)	<0.001

BMI: body mass index; ESS: Epworth Sleepiness Scale; FFM: fat free mass; FM: fat mass; MEQ: Horne–Östberg Morningness–Eveningness questionnaire; MVPA: moderate to vigorous physical activity; NW: normal weight controls; OB: adolescents with obesity; PSQI: Pittsburgh sleep quality index; RMR: resting metabolic rate; SD: standard deviation; TEE: Total energy expenditure.

**Table 2:** Sleep parameters among adolescents with obesity and their matched normal weight controls during 3rd night of ad libitum and eucaloric sessions.

	OB		NW		ANOVA					
	Ad libitum	Eucaloric	Ad libitum	Eucaloric	Interaction (Weight Status × Condition)		Weight Status Effect		Condition Effect	
	Mean (SD)	Mean (SD)	Mean (SD)	Mean (SD)	F	*p* Value	F	*p* Value	F	*p* Value
SE (%)	77.0 (9.6)	78.0 (10.4)	90.5 (6.3)	91.1 (6.7)	0.367	0.550 ES = 0.24	17.310	**<0.001** ES = 1.64	5.591	**0.026** ES = 0.92
SL (min)	32.0 (24.8)	23.5 (21.6) **	19.7 (11.8)	16.1 (13.0)	3.699	0.065 ES = 0.76	2.012	0.168 ES = 0.56	22.576	**<0.001** ES = 1.86
WASO (min)	92.0 (46.3)	95.0 (49.8)	31.5 (23.8)	31.9 (25.3)	1.216	0.280 ES = 0.44	18.420	**<0.001** ES = 1.68	1.962	0.173 ES = 0.54
Wake (min)	124.0 (51.8)	118.6 (56.5)	51.2 (34.3)	48.0 (36.6)	0.367	0.550 ES = 0.24	17.310	**<0.001** ES = 1.64	5.591	**0.026** ES = 1.92
Stage REM (min)	74.1 (26.9)	76.0 (26.5)	103.5 (40.8)	95.8 (38.3)	3.149	0.088 ES = 0.70	3.873	0.060 ES = 0.78	1.199	0.280 ES = 0.42
Stage N1 (min)	36.6 (9.4)	30.6 (11.8) *	30.7 (16.3)	29.7 (17.8)	5.508	**0.027** ES = 0.92	0.421	0.522 ES = 0.24	10.797	**0.003** ES = 1.28
Stage N2 (min)	209.0 (29.1)	209.7 (33.1)	256.7 (46.1)	258.2 (48.6)	0.883	0.356 ES = 0.36	10.838	**0.003** ES = 1.3	0.044	0.836 ES = 0.08
Stage N3 (min)	96.0 (45.0)	105.0 (54.5)	97.7 (47.7)	108.0 (50.3)	0.034	0.856 ES = 0.06	0.002	0.961 ES < 0.01	6.434	**0.018** ES = 1.00
Arousals (events/h)	17.3 (2.3)	15.4 (3.7)	13.3 (5.4)	12.34 (3.68)	0.637	0.432 ES = 0.32	6.502	**0.017** ES = 1.00	7.099	**0.013** ES = 1.04

Eucaloric: dietary intake to energy requirement; NW: normal-weight controls; OB: adolescents with obesity; REM: rapid eye movement; SE: sleep efficiency; SL: sleep latency; SD: standard deviation; significant *p* values are bolded; *: significant difference in post hoc pairwise comparisons (ad libitum vs. eucaloric) with *p* < 0.05. **: significant difference in Post hoc pairwise comparisons (ad libitum vs. eucaloric) with *p* < 0.01. ES: effect size.

## Data Availability

The data presented in this study are available on request from the corresponding author and the permission of all parties involved in the study.

## Supplementary Materials

The following are available online at https://www.mdpi.com/article/10.3390/nu13103550/s1, Table S1: Food items included in the ad libitum buffet meals and the standardized eucaloric meals, Table S2: Energy derived from each macronutrient among adolescents with obesity and their matched normal weight controls during *ad libitum* and eucaloric sessions, Table S3: Energy derived from each macronutrient among adolescents with obesity (excluding the 4 participants with OSA) and their matched normal weight controls during ad libitum and eucaloric sessions, Table S4: Distribution of energy intake throughout the four meals among adolescents with obesity (excluding the 4 participants with OSA) and their matched normal weight controls during ad libitum and eucaloric sessions, Table S5: Sleep based on EEG-recording among adolescents with obesity (excluding the 4 participants with OSA) and their matched normal weight controls during 3rd night of ad libitum and eucaloric sessions. Figure S1: Heatmap representation of the correlations between Δ energy intake and Δ sleep outcomes between sessions (ad libitum–eucaloric) in NW group.
