# Supplementary material for: Eucaloric Balanced Diet Improved Objective Sleep in Adolescents with Obesity
Source: Nutrients. 2021 Oct 10;13(10):3550. doi: 10.3390/nu13103550 (PMC8537106; doi:10.3390/nu13103550)
Supplement: Supplementary file 1 [file nutrients-13-03550-s001.zip › nutrients-1371188-supplementary.pdf]

Table S1: Food items included in the standardized and *ad libitum* buffet meals

	Groups	Food	Nutritional values per 100g of food
Breakfast	Dairy products	1 Bowl of milk (250mL)	116 kcal
		1 Yogurt (125g)	78 kcal
	Fruits	1 Orange or 1 apple or 1 pear	67 kcal
	Grain (cereal)	Bread (80g)	210 kcal
		Cereal grains (60g)	231 kcal
	Sweet product, sugars	Apricot jam (10g)	26 kcal
	Fats	Butter (10g)	74 kcal
	Vegetables and legumes (raw)	Grated carrot	32 kcal
	Fish	White fish fillet	154 kcal
	Grain (cereal)	Steamed rice	133 kcal
Lunch	Vegetables and legumes/beans (cooked)	Mixed vegetables (onion, carrot, tomatoes, red and yellow bell pepper)	117 kcal
	Dairy products	Unflavoured yoghurt (125g)	62 kcal
	Sweet product, sugars	Applesauce (100g)	90 kcal
		Sugars (5g)	398 kcal
Dinner	Vegetables and legumes (raw)	Coleslaw	26 kcal
	Lean meats and poultry	Sliced turkey	121 kcal
	Grain (cereal)	Cooked pasta	145 kcal
		bread	263 kcal
	Vegetables and legumes/beans (cooked)	French bean	27 kcal
	Dairy products	Cottage cheese	74 kcal
	Fruits	1Seasonal fruit	48 kcal
Snack	Dairy products	1 Bowl of milk (250mL per serving)	116 kcal
		1 Yogurt (125g per serving)	78 kcal
		Brie cheese (20g per serving)	334 kcal
	Fruits	1 Orange or 1 apple or 1 pear (1 fruit per serving)	67 kcal
		Orange juice (100 mL per serving)	45kcal
	Grain (cereal)	Bread (80g per serving)	210 kcal
		cereal grains (60g per serving)	231 kcal

Nuts and Seeds	Walnuts (3 per serving)	654 kcal
Sweet product, sugars	Apricot jam (10g per serving)	242 kcal
	Honey (10g per serving)	304 kcal
	Peaches canned in syrup	96 kcal
Fats	Butter (10g per serving)	74 kcal

Table S2. Energy derived from each macronutrient among adolescents with obesity and their matched normal weight controls during *ad libitum* and eucaloric sessions

	OB		NW		ANOVA					
	Ad libitum	Eucaloric	*Ad libitum*	Eucaloric	Interaction (Weight status × condition)		Weight status effect		Condition effect	
	**Mean (SD)**	**Mean (SD)**	**Mean (SD)**	**Mean (SD)**	**F**	**P value**	**F**	**P value**	**F**	**P value**
Protein (%EI)	15.12 (1.74)	18.01 (0.29)	20.09 (3.15)	18.01 (0.36)	11.48	**<0.001** ES=1.94	13.51	**<0.001** ES=2.10	0.57	0.456 ES=0.28
Lipids (%EI)	30.52 (2.59)	30.05 (0.42)	30.06 (3.60)	30.16 (0.45)	0.169	0.845 ES=0.24	0.069	0.934 ES=0.20	0.264	0.612 0.14
CHO (%EI)	54.35 (1.97)	51.94 (0.38)	49.85 (3.96)	51.82 (0.56)	6.04	**0.007** ES=1.32	7.40	**0.003** ES=1.44	0.011	0.917 ES=0.08

CHO: carbohydrates; EI: energy intake; ES: effect size; NW: normal weight controls; OB: adolescents with obesity; significant P values are bolded.

Table S3. Energy derived from each macronutrient among adolescents with obesity (excluding the 4 participants with OSA) and their matched normal weight controls during *ad libitum* and eucaloric sessions

	**OB**		**NW**		**ANOVA**					
	Ad libitum	**Eucaloric**	***Ad libitum***	**Eucaloric**	**Interaction** (Weight status × condition)		**Weight status effect**		**Condition effect**	
	**Mean (SD)**	**Mean (SD)**	**Mean (SD)**	**Mean (SD)**	**F**	**P value**	**F**	**P value**	**F**	**P value**
Protein (%EI)	15.29 (1.70)	17.93 (0.27)	20.09 (3.15)	18.01 (0.36)	17.25	<0.001 ES=1.08	21.94	<0.001 ES=1.13	0.21	0.650 ES=0.11
Lipids (%EI)	30.66 (2.83)	30.05 (0.42)	30.00 (3.55)	30.14 (0.46)	0.30	0.589 ES=0.17	0.20	0.658 ES=0.13	0.10	0.753 0.09
CHO (%EI)	54.05 (2.17)	52.02 (0.23)	49.90 (3.99)	51.85 (0.57)	7.70	0.001 ES=1.77	9.79	0.005 ES=0.85	0.003	0.955 ES<0.001

CHO: carbohydrates; EI: energy intake; ES: effect size; NW: normal weight controls; OB: adolescents with obesity; OSA: obstructive sleep apnea; significant P values are bolded.

Table S4. Distribution of energy intake throughout the four meals among adolescents with obesity (excluding the 4 participants with OSA) and their matched normal weight controls during ad libitum and eucaloric sessions

	**OB without OSA**		**NW**		**ANOVA**					
	Ad libitum	**Eucaloric**	***Ad libitum***	**Eucaloric**	**Interaction (Weight status × condition)**		**Weight status effect**		**Condition effect**	
	**Mean (SD)**	**Mean (SD)**	**Mean (SD)**	**Mean (SD)**	**F**	**p value**	**F**	**p value**	**F**	**p value**
EI Dinner	942.20(155.47)	848.50(128.73)	729.64(95.79)	730.36(46.70)	6.63	**0.017** ES=0.35	16.40	**<0.001** ES=1.50	6.43	**0.019** ES=0.35
EI Lunch	1347(173.23)	855.50(126.41)	803.86(107.14)	735(51.95)	67.88	**<0.001** ES=0.86	65.82	**<0.001** ES=1.59	119.32	**<0.001** ES=1.23
EI breakfast	781.30(123.56)	705.30(106.07)	644.71(46.87)	609.29(41.34)	4.71	**0.041** ES=0.20	13.001	**0.002** ES=1.38	35.53	**0.001** ES=0.57
EI snacks	468.90(81.30)	429.90(65.20)	372.79(21.91)	350.57(50.10)	0.93	0.345 ES=0.11	16.85	**<0.001** ES=1.56	12.39	**0.002** ES=0.44

EI: energy intake; ES: effect size; NW: normal weight controls; OB: adolescents with obesity; OSA: obstructive sleep apnea; significant P values are bolded.

Table S5. Sleep based on EEG-recording among adolescents with obesity (excluding the 4 participants with OSA) and their matched normal weight controls during 3rd night of *ad libitum* and eucaloric sessions.

	OB without OSA		NW		ANOVA					
	Ad libitum	Eucaloric	*Ad libitum*	Eucaloric	Interaction (weight status × condition)		Weight status effect		Condition effect	
	Mean (SD)	Mean (SD)	Mean (SD)	Mean (SD)	F	p value	F	p value	F	p value
SE (%)	78.17 (10.38)	79.74 (11.21)	90.50 (6.37)	91.10 (6.78)	1.71	0.204<br>ES=0	11.30	**0.003**<br>ES=1.42	8.39	**0.008**<br>ES=0.11
SL (min)	30.70 (23.30)	20.20 (18.07)	19.71 (11.89)	16.14 (13.09)	6.22	**0.021**<br>ES=0.21	1.27	0.271<br>ES=0.46	25.67	**<0.001**<br>ES=0.43
WASO (min)	87.20 (46.73)	89.20 (51.16)	31.57 (23.86)	31.93 (25.35)	0.36	0.557	13.99	**0.001**<br>ES=1.59	0.73	0.402
Wake (min)	117.90 (56.05)	109.40 (60.52)	51.29 (34.37)	48.07 (36.61)	1.71	0.204	11.30	**0.003**<br>ES=1.42	8.41	**0.008**<br>ES=0.11
Stage REM (min)	73.91 (29.87)	75.40 (27.13)	103.50 (40.83)	95.86 (38.32)	2.35	0.140	3.03	0.096	1.06	0.314
Stage N1 (min)	38.10 (8.06)	30.70 (8.95)	30.71 (16.35)	29.71 (17.82)	9.09	**0.006**<br>ES=0.23	0.52	0.478	15.66	**<0.001**<br>ES=0.3
Stage N2 (min)	211.70 (23.73)	206.80 (26.27)	256.79 (46.18)	258.29 (48.65)	3.01	0.097	8.69	**0.007**<br>ES=1.25	0.85	0.366
Stage N3 (min)	98.39 (52.95)	117.70 (59.59)	97.71 (47.71)	108.07 (50.39)	0.87	0.362	0.06	0.809	9.52	**0.005**<br>ES=0.29
Arousals (events/h)	18.04 (2.33)	14.94 (4.26)	13.38 (5.47)	12.34 (3.68)	3.12	0.091	4.90	**0.038**<br>ES=0.86	12.51	**0.002**<br>ES=0.46

Eucaloric: dietary intake to energy requirement; NW: normal-weight controls; OB: adolescents with obesity; OSA: obstructive sleep apnea; REM: rapid eye movement; SE: sleep efficiency; SL: sleep latency; SD: standard deviation; significant P values are bolded; *: significant difference in Post hoc pairwise comparisons (*ad libitum* vs eucaloric) with p<0.05. **: significant difference in Post hoc pairwise comparisons (*ad libitum* vs eucaloric) with p<0.01. ES: effect size.

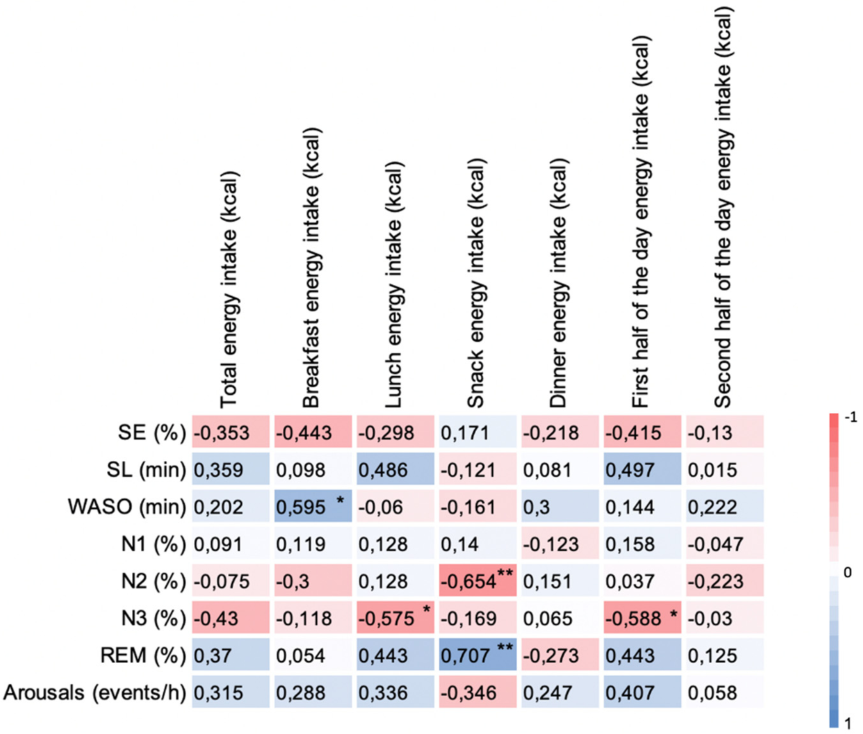

Figure S1. Heatmap representation of the correlations between Δ energy intake and Δ sleep outcomes between sessions (*ad libitum* – eucaloric) in NW group. Red indicates a negative relationship whereas blue indicates a positive relationship; the darker the color, the higher the Pearson coefficient; *: significant correlation with $p<0.05$; **: significant correlation with $p<0.01$.
